# Phase‐Engineered Non‐Degenerate Sliding Ferroelectricity Enables Tunable Photovoltaics in Monolayer Janus In_2_S_2_Se

**DOI:** 10.1002/advs.202520732

**Published:** 2025-12-14

**Authors:** Yixuan Li, Qiang Wang, Keying Han, Yitong Liang, Kai Kong, Yan Liang, Thomas Frauenheim, Xingshuai Lv, Defeng Guo, Bin Wang

**Affiliations:** ^1^ State Key Laboratory of Metastable Materials Science and Technology and Key Laboratory for Microstructural Material Physics of Hebei Province School of Science Yanshan University Qinhuangdao 066004 P.R. China; ^2^ College of Physics and Optoelectronic Engineering Faculty of Information Science and Engineering Ocean University of China Songling Road 238 Qingdao 266100 P.R.China; ^3^ School of Science Constructor University 28759 Bremen Germany; ^4^ Computational Science and Applied Research Institute (CSAR) Shenzhen 518110 P. R. China; ^5^ Beijing Computational Science Research Center (CSRC) Beijing 100193 P. R. China; ^6^ College of Chemistry and Chemical Engineering Ocean University of China Qingdao 266100 P. R. China; ^7^ Shenzhen Key Laboratory of Advanced Thin Films and Applications College of Physics and Optoelectronic Engineering Shenzhen University Shenzhen 518060 P.R. China

**Keywords:** 2D non‐degenerated sliding ferroelectricity, 2D photoelectronic devices, 2D photovoltaic modulation, first‐principles calculations, monolayer In2S2Se Phase transition

## Abstract

2D sliding ferroelectrics, with their enhanced efficiency of charge separation and tunability, provide promising platforms for next‐generation photovoltaic devices. However, recent systems predominantly exhibit dual degenerate polarization states with weak intensity, limiting the optimal manipulations of photovoltaic effects through sliding ferroelectricity. Here, this issue is addressed by introducing two strengthened and distinct non‐degenerate sliding ferroelectric states (*WZ*′ and *ZB*′) in Janus *In*_2_ *S*_2_ *Se*, which can be achieved by Se‐to‐S substitution in monolayer *In*_2_ *Se*_3_. First–principles calculations demonstrate the experimental feasibility and reversible transition between these states triggered by atomic layer sliding. Remarkably, the *WZ*′‐to‐*ZB*′ switch enhances carrier mobility, reduces photogenerated carrier lifetimes, narrows the bandgap with an indirect‐to‐direct transition, and induces a pronounced redshift and photocurrent enhancement in the infrared region. Conversely, the *WZ*′ state, with stronger polarization, achieves higher photoelectric conversion efficiency under visible light. This work establishes a state‐engineered framework of how non‐degenerate sliding ferroelectricity orchestrates distinct photovoltaic behaviors, and the intrinsic physical correlations may offer novel perspectives for next designing and regulating innovative photovoltaic devices.

## Graphical abstract

The meticulously phase‐engineered non‐degenerate sliding ferroelectricities in monolayer *In*_2_ *S*_2_ *Se*, along with their tunable impacts on photovoltaic performances, introduce innovative perspectives for the future design and optimization of 2D advanced photovoltaic devices.

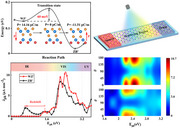

## 1 Introduction

2D excitonic solar cells (2D XSCs), due to its intrinsic potential for low‐cost, eco‐friendly, and highly efficient photoelectric conversion surpass the conventional bulk ones, is considering as ideal candidates to address recent environmental and energy crises.^[^ ^1^ ^]^ In these thin films, photo‐generated carriers are generated upon illumination, and then separated under varying interfacial electron ionization and affinity potentials. To date, the rapid exciton recombination remains a critical bottleneck limiting the power‐conversion efficiency in 2D XSCs.^[^ ^2^ ^]^ One promising strategy is integrating ferroelectricity and photovoltaics into a single system,^[^ ^3^ ^]^ in which the spontaneous out‐of‐plane polarization (OOP) can effectively inhibit carrier recombination, thereby improving their photoelectric conversion efficiencies.^[^ ^4^, ^5^ ^]^ Recent theoretical and experimental evidences have also demonstrated this assertion.^[^ ^6^, ^7^ ^]^ So far, a class of molecule ferroelectrics has been proposed, especially in the molecule ferroelectric,^[^ ^8^ ^]^ with robust room‐temperature ferroelectricity in their van der Waals (vdW) ferroelectrics can be maintained in sub‐10 nm thickness.^[^ ^9^ ^]^ However, naturally occurring 2D ferroelectric materials are exceedingly rare due to the stringent symmetry requirements.^[^ ^10^ ^]^ Exploring novel formation mechanisms of 2D ferroelectricity and deciphering its role in amplifying photovoltaic responses are essential to enhance the photovoltaic metrics in next‐generation of 2D XSCs.

2D sliding ferroelectrics, with the OOP polarization generated by interlayer asymmetric stacking, enabling nonpolar monolayers to acquire switchable dipoles through interlayer sliding, thus can vastly broadens the pool of 2D ferroelectrics.^[^ ^11^ ^]^ Such concept is initially proposed theoretically in 2017,^[^ ^12^ ^]^ and has been experimentally implemented since 2021.^[^ ^11^, ^13^ ^]^ Owing to the robust structure, easy experimental operation, high Curie temperatures and fatigue‐free switching,^[^ ^14^ ^]^ they are attractive for memory and actuator applications.^[^ ^15^ ^]^ Besides, the built‐in polarization fields in these materials can drive superior photovoltaic effects, enabling enhanced charge separation,^[^ ^16^ ^]^ intrinsic type‐II band alignments,^[^ ^17^ ^]^ above–bandgap photovoltages^[^ ^18^ ^]^ and enhanced photoelectric conversion efficiencies over the limitation of Shockley‐Queisser^[^ ^19^ ^]^ in 2D devices. Despite these advances, significant challenges remain in realizing ideal 2D sliding ferroelectric photovoltaic systems: One is the insufficient polarization magnitudes resulting from weak interlayer vdW coupling,^[^ ^20^ ^]^ which impedes the efficient separation of photo‐generated carriers; The other refers to the presence of dual degenerate polarization states,^[^ ^20^, ^21^ ^]^ impossible to explore the intrinsic correlations between ferroelectricity and photovoltaic effects. Recently, various extrinsic modulations have been proposed for the optimal tune of their interlayer polarization, such as strain.^[^ ^22^ ^]^ thickness^[^ ^23^ ^]^ and so forth. However, these external approaches often induce structural distortions, obscuring the intrinsic role of polarization in photoelectric processes. Further efforts should be focus on the materials design and scalable assembly techniques to unlock the full potential of sliding ferroelectrics in photovoltaics.

Monolayer α − *In*_2_ *Se*_3_, featured by a corrugated quintuple‐layer honeycomb structure composed of alternating Se–In–Se–In–Se atomic layers, is an ideal representative that integrates ferroelectricity and photovoltaics.^[^ ^6^, ^24^ ^]^ Since the built‐in asymmetry breaks its OOP inversion symmetry, enabling robust spontaneous OOP polarization in this emerging monolayer, which is still switchable through ionic displacement of the middle Se atom layer.^[^ ^24^, ^25^ ^]^ Recent theoretical and experimental studies have demonstrated series its photovoltaic superiorities, including the narrow bandgap (≈1.45 eV),^[^ ^26^ ^]^ the above–band‐gap photovoltages,^[^ ^7^ ^]^ and the photocurrents two orders of magnitude higher than bulk ferroelectrics.^[^ ^27^ ^]^ The ultrafast and nonvolatile photocurrent hysteresis^[^ ^6^ ^]^ further highlights its potential for integrated optoelectronics and all‐optical signal processing. More inspiringly, this special material is also confirmed to share two distinct ground states, namely *ZB*′ and *WZ*′,^[^ ^24^ ^]^ offering more versatile ferroelectric characteristics for photovoltaic modulation.

Janus monolayers, with the two faces bear different atomic species or chemical terminations, exhibit stronger OOP dipoles due to the intensified broken of inversion symmetry.^[^ ^28^ ^]^ This asymmetric structure is initially proposed theoretically based on transition‐metal dichalcogenides in 2013,^[^ ^29^ ^]^ which have been experimentally realized by selective replacement of one chalcogen face,^[^ ^30^ ^]^ synthetic routes now extend to oxides,^[^ ^31^ ^]^ halides,^[^ ^32^ ^]^ and rare–earth compounds.^[^ ^33^ ^]^ Due to the spontaneous and switchable OOP polarization, such specific monolayers have confirmed a series of outstanding performances, including the ferroelectric non‐volatile storage,^[^ ^34^ ^]^ photovoltaics,^[^ ^35^ ^]^ and photocatalysis.^[^ ^36^ ^]^ Inspired by these systems, the Janus α − *In*_2_ *Se*_3_ can be realized by substituting layers of Se atoms with the homotopic S. Compared to pristine α − *In*_2_ *Se*_3_, this Janus one ought to exhibit enhanced charge separation capabilities due to the asymmetrical bilateral atoms and stronger OOP dipole. In addition, such asymmetric structure also gives rise to two non‐degenerate polarization states between *ZB*′ and *WZ*′, enable more adjustable polarization intensities and photovoltaic characters during their sliding transition. Therefore, we can wonder more controllable and superior photovoltaic performances in this newly predicted Janus monolayer, and further exploring the underlying correlation mechanisms between 2D sliding ferroelectricity and photovoltaics.

Here, the *In*_2_ *S*_2_ *Se* is chosen because of its optimal bandgap and superior carrier mobility among Janus various *In*_2_ *Se*_3_ monolayers.^[^ ^37^ ^]^ First‐principles calculations confirm its structural, dynamic and thermal stability. Compared to the pristine α − *In*_2_ *Se*_3_, its enhanced bilateral asymmetry yields stronger out‐of‐plane polarization and more efficient charge separation, driving improved photovoltaic performance. More strikingly, we demonstrate two non‐degenerate sliding‐ferroelectric phases, *ZB*′ and *WZ*′, which can be reversible via interlayer sliding at experimentally accessible energy barriers. Further detailed analysis of its regulation effects on photovoltaics is conducted, where the *WZ*′‐to‐*ZB*′ transition red‐shifts and amplifies the infrared photocurrent peak, while the *WZ*′ phase delivers higher conversion efficiency under visible illumination. Together, these results establish a phase‐engineered link between 2D sliding ferroelectricity and tunable photovoltaics, paving the way for next‐generation 2D optoelectronic devices.

## 2 Model and Numerical Method

For the periodic system of Janus *In*_2_ *S*_2_ *Se*, the crystal relaxation, structural stabilities, electronic properties, and light absorption coefficients were simulated by using the Vienna ab initio simulation package (VASP), which is based on density functional theory (DFT).^[^ ^38^ ^]^ The exchange correlation functional of Perdew–Burke–Ernzerhof (PBE) level was used to deal with the geometrical optimization and electronic structure self‐consistent calculations within the generalized gradient approximation (GGA).^[^ ^39^ ^]^ To address the underestimation of bandgap at PBE level, the more accurate results were calculated based on the hybrid density functional of Heyd–Scuseria–Ernzerhof (HSE06), and the Hartree–Fock exchange energy was set 25%.^[^ ^40^ ^]^ To expand the electron wave function into plane waves, the projector augmented wave (PAW) approach was employed with a plane wave cut off energy of 500 eV.^[^ ^41^ ^]^ The convergence criteria were 0.01 *eV* *Å* ^−1^ for force and 10^−5^ eV for energy, a k‐point mesh of 15 × 15 × 1 was used to sample the Brillouin zone, and a vacuum larger than 20 Å was used to eliminate the spurious interactions perpendicular to the 2D plane. In addition, the dynamic stability for each phase was confirmed by the phonon spectrum, which was calculated by using the Nanodcal code. The thermal stability was verified by performing an ab initio molecular dynamics (MD) simulation within a 3 × 3 × 1 supercell under 300 K, the evolution time was set 5 ps with a time step of 1 fs. The energy barriers during the sliding ferroelectric switching were calculated under the method of nudged elastic band (NEB), with the direction and intensity of OOP polarization were evaluated combining the approaches of voltage drop and Bader charge analysis.

By using the Hefei‐NAMD code,^[^ ^42^, ^43^ ^]^ the nonadiabatic molecular dynamics (NAMD) simulations were conducted to investigate the thermal stability and lifetimes of photo‐generated carriers for *In*_2_ *S*_2_ *Se*. To first evaluate the thermal stability, ab initio molecular dynamics (AIMD) simulations were performed on a 3 × 3 × 1 supercell at 300 K within the NVT ensemble for 5 ps, using a time step of 1 fs. Subsequently, an adiabatic MD trajectory of 10 ps was performed still with a time step of 1 fs in the microcanonical ensemble (NVE). Finally, 1000 initial configurations combined with 2000 surface‐hopping trajectories were sampled for each configuration to perform long‐timescale NAMD simulations.

The simulations of In_2_S_2_Se based two‐probe nano‐devices were performed by using the first‐principles quantum transport package Nanodcal,^[^ ^44^, ^45^ ^]^ which is based on the combination of non‐equilibrium Green's function (NEGF) and density functional theory (DFT).^[^ ^44^ ^]^ During our calculation, the generalized gradient approximation at PBE level was used to handle the exchange‐correlation potential, the wave functions were expanded by the basis sets using atomic orbitals of double‐zeta polarization (DZP), the norm‐conserving non‐local pseudo‐potential was applied to define the atomic core, and the energy convergence criterion of self‐consistence was set to be less than 10^−5^ eV.^[^ ^38^ ^]^ Besides, a k‐mesh grid of 64 × 1 for center and 256 × 1 for the leads were set perpendicular to the transport direction during the self‐consistent and photocurrent computations.

## 3 Numerical Results and Discussion

### 3.1 Structure, Stability, and OOP Polarization of *In*_2_S_2_ *Se*

**Figure 1:**
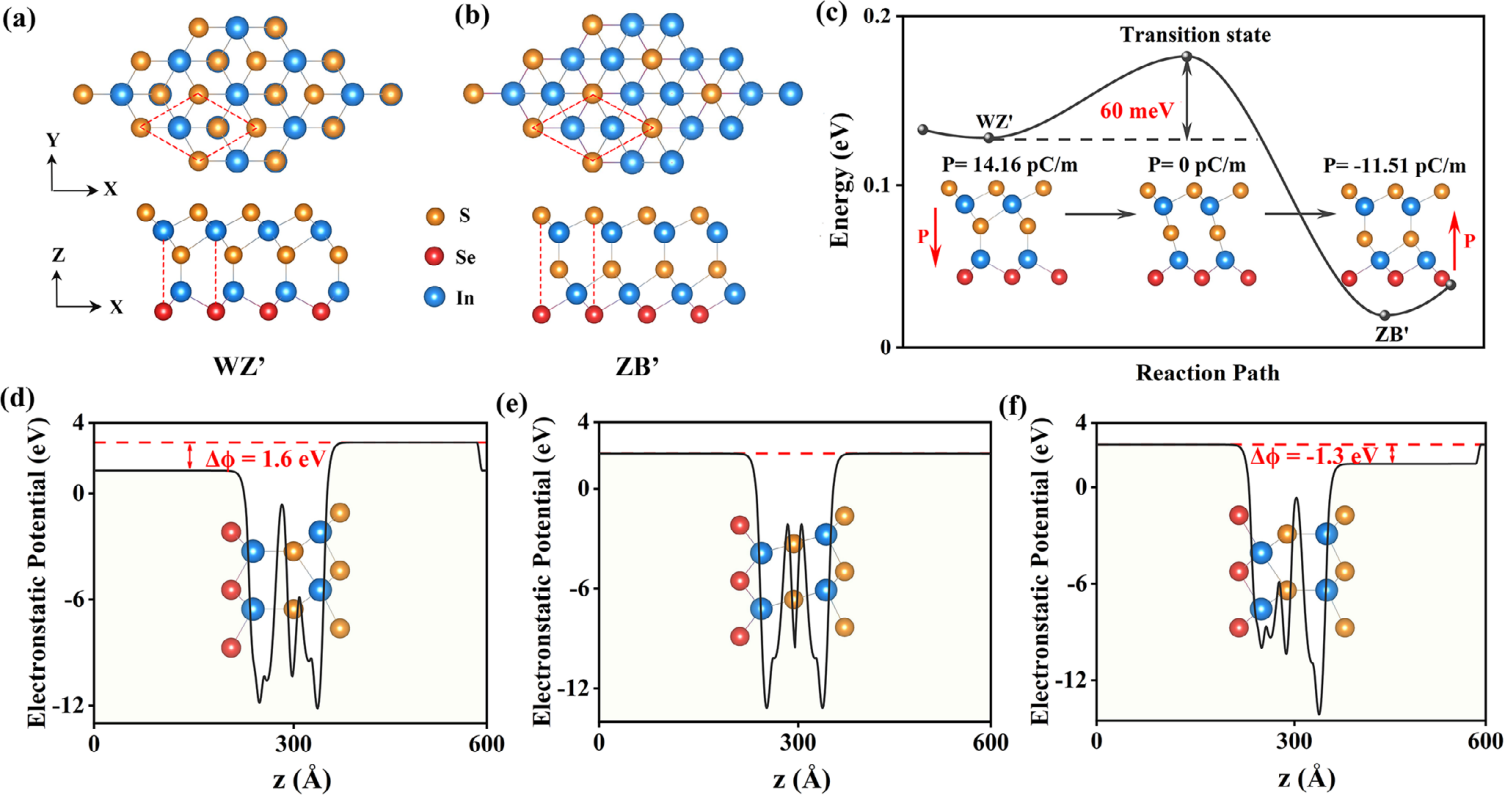
a,b)Top and side views of schematic structures of monolayer *In*_2_S_2_ *Se* under *WZ*′ (a) and *ZB*′ (b) states. In each panel, the red diamond represents the size of one unit cell, with S, Se, and In atoms distinguished by yellow, green, and purple spheres, respectively. c) Energy variation of *In*_2_S_2_ *Se* along the out‐of‐plane ferroelectric switching pathway under NEB simulation. The three inserts depict configurations at the initial, middle, and final states, with *P* indicating the corresponding OOP polarization strength. d–f) Plane‐averaged electrostatic potentials along the *Z* axis for *In*_2_S_2_ *Se* at different states.

The fully optimized In_2_S_2_ *Se* structures under *WZ*′ (a) andS *ZB*′ (b) states are displayed in **Figure** **1**a,b, where two Se atomic layers are substituted by the homotopic S. According to the different layers of atomic substitution, each state exhibits three distinct configurations: *t* − *In*_2_S_2_ *Se*, *m* − *In*_2_S_2_ *Se*, and *b* − *In*_2_S_2_ *Se* (Figure S1, Supporting Information). To obtain the most stable configuration, the binging energy for each structure is calculated using the formula: *E*_b_ = (*E*_total_ − *nE*_In_ − *mE*_S_ − *kE*_Se_)/(*m* + *n* + *k*), where *E*_*total*_, *E*_*In*_, *E*_*S*_, and *E*_*Se*_ represent the energies of monolayer material, a single In atom, S atom and Se atom. As shown in Table S1, the *E*_*b*_ of different stacking In_2_S_2_ *Se* can be reached 1.1 − −1.2*Jm* ^2^, larger than the typical α − *In*_2_ *Se*_3_, indicating their energy feasibility. Since *b*‐In2S2Se possesses the lowest *E*_*b*_ among all configurations in each state, these two configurations are chosen for subsequent analysis. To examine the experimental feasibility of these two *In*_2_S_2_ *Se* monolayers, their phonon spectrum and ab initio molecular dynamics simulations are conducted in Figure S2 (Supporting Information). First, the phonon spectra contains no virtual frequencies over the entire Brillouin zone, indicating stable minimum values of these two monolayers on potential energy, hence both of them are all dynamically stable.^[^ ^46^ ^]^ Additionally, to explore the thermal stability of these two monolayers, the temporal evolution of their total energy are also simulated in Figure S2 c,d (Supporting Information). For ZB' phase, the total energy fluctuates around a constant value with minimal amplitude, while in *WZ*′ phase, the total energy gradual increases with just minimum magnitude during the temporal evolution. For both phases, only slight changes in atomic configurations occur after 5 ps of evolution, with no obvious geometry reconstruction or bond breaking. All these features confirm that both phases of the In_2_S_2_ *Se* monolayer are thermal stability at the room temperature. These stability characteristics suggest robust feasibility for the experimental preparation of both *WZ*′ and *ZB*′ In_2_S_2_ *Se*, and provide solid basis for their further sliding ferroelectric and photovoltaic related characters investigation.

Inspired by the sliding ferroelectricity in monolayer In_2_Se_3_, we wonder whether it can be maintained in the Janus In_2_S_2_ *Se*. As illustrated in Figure 1c, via specific middle and bottom atomic layers sliding, the phase transition between *WZ*′ and *ZB*′ states can be achieved in this Janus In_2_S_2_ *Se*. The evolution energy barrier from *WZ*′‐to‐*ZB*′ is ≈ 60*meV*, and the reverse *ZB*′ to *WZ*′ barrier is higher to 97 meV, both are experimentally achievable and closed to that of monolayer In_2_Se_3_ (66 meV)^[^ ^24^ ^]^. Owing to the intrinsic atomic inherent to its dual‐sided composition, enhanced OOP polarization intensities can be detected in such Janus In_2_S_2_ *Se*, which can be reached $P_{WZ^{\prime }}$ = 14.16 *pCm* ^−1^ under *WZ*′ state and $P_{ZB^{\prime }}$ = ‐11.51 *pCm* ^−1^ under *ZB*′ state, higher than the typical monolayer α − *In*_2_ *Se*_3_ ($P_{WZ^{\prime }}$ = $P_{ZB^{\prime }}$ = 10 *pCm* ^−1^),^[^ ^47^ ^]^ under identical computational conditions. Accordingly, we can predict that the enlarged transition barrier from *ZB*′ to *WZ*′ phase is originating from their non‐degeneracy rather than the OOP polarization. Beside significant stronger OOP polarization can also be confirmed in this Janus monolayer than the bilayer BN (2.0 *pCm* ^−1^)^[^ ^11^ ^]^ and MoS_2_(2.2 *pCm* ^−1^).^[^ ^48^ ^]^

To verify the distinct directions and intensities OOP polarization of this Janus In_2_S_2_ *Se*, the electrostatic potentials under different states along Z axis are displayed in Figure 1d–f. The potential energy decrease ΔΦ are 1.6 eV for *WZ*′ state and ‐1.3 eV for *ZB*′ state, which is non‐degenerated and higher than those of monolayer α − *In*_2_ *Se*_3_, reinforcing the stronger and non‐degenerate OOP sliding ferroelectricity. Interestingly, not only such enhanced OOP polarization in Janus In_2_S_2_ *Se* may resulting in superior photovoltaic performances, the non‐degenerate OOP ferroelectric states can also provide a new regulated strategy of sliding ferroelectric on photovoltaics.

### 3.2 Photovoltaics Related Electronic Characters of *In*_2_S_2_ *Se*

**Figure 2:**
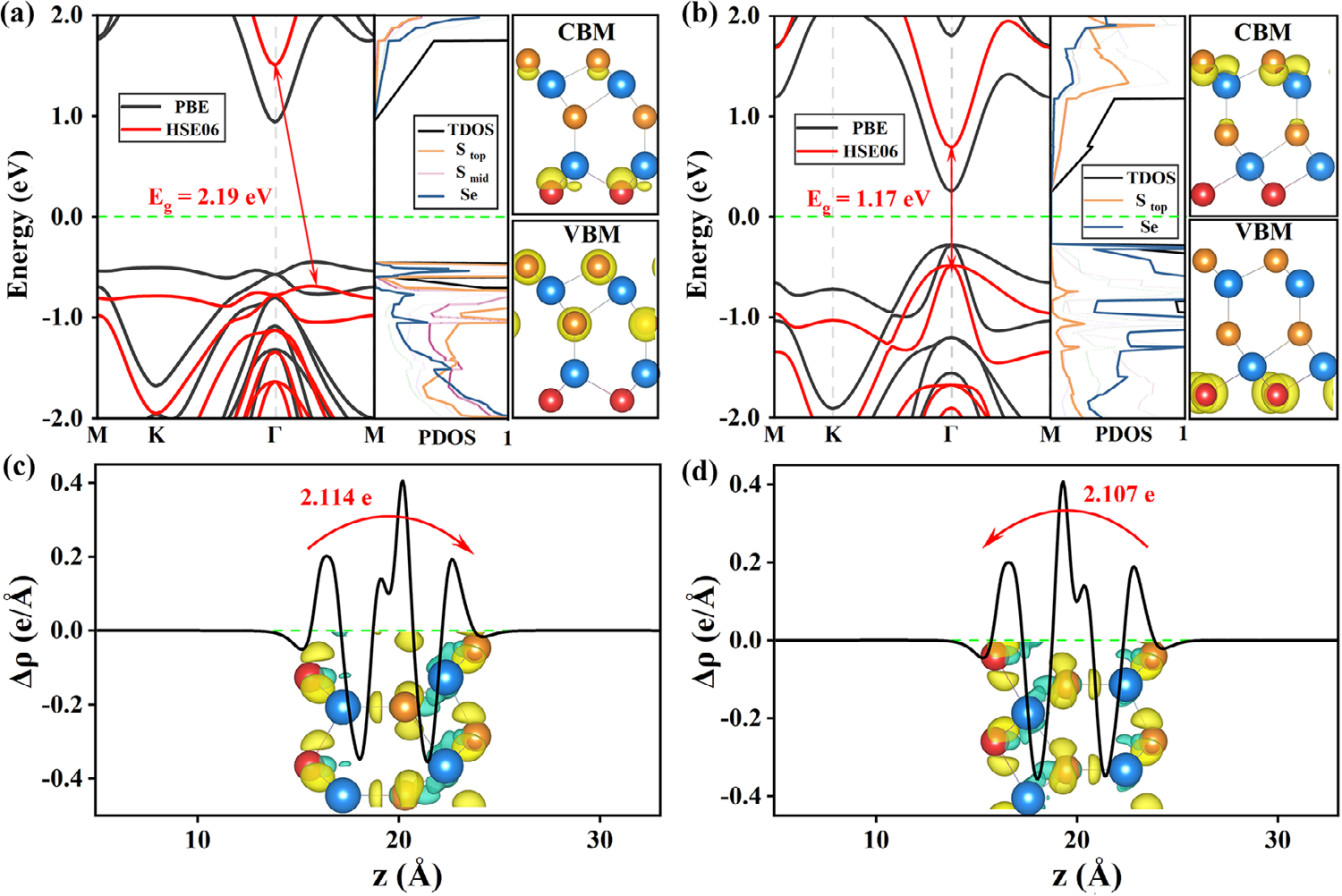
a,b) The calculated band structures (left panel), PDOSs (middle panel), and partial charge densities at CBM and VBM (right panel) for *In*_2_S_2_ *Se* under *WZ*′ (a) and *ZB*′ (b) states. In each panel, the band curvess at PBE and HSE06 levels are distinguished by black and red lines, the fermi level is set to zero and marked by the green dashed line, and the isosurface value in the right panel is set at 0.015*e* *Å* ^−3^. c,d) 3D isosurface and 2D integrated charge density differences along *Z* axis of *In*_2_S_2_ *Se* under *WZ*′ (c) and *ZB*′ (d) states, with isosurface value being set at 0.005*e* *Å* ^−3^, the charge accumulation and dissipation are indicated by green and orange balls, respectively.

Next, the electronic properties between *WZ*′ and *ZB*′ In_2_S_2_Se are investigated in‐depth to explore their photovoltaic superiority. First, the band structures, projected density of states (PDOS) and partial charge densities are comparatively studied in **Figure** **2**a,b. Obviously, with the sliding phase transition from *WZ*′ to *ZB*′, an indirect‐to‐direct behavior occurs accompanied by a decrease of bandgap. When in *WZ*′ state, the bandgap are 2.19 (1.42) eV at HSE06 (PBE) level, which is too larger for visible‐light absorption,^[^ ^49^ ^]^ let alone the non‐beneficial of indirect bandgap on the production of photogenerated charge carriers.^[^ ^50^ ^]^ Inspiringly, when the phase is transferred into the *ZB*′, a moderate direct band gap of 1.17 (0.54) eV at HSE06 (PBE) level can be obtained, which perfectly fits the optimum range for excitonic solar cells (1.2–1.6 eV).^[^ ^51^ ^]^ Another critical factor of 2D photovoltaic candidates is the capabilities of real space charge separation. As shown in the middle and right panels of Figure 2a,b, obvious charge separation behaviors can be observed in both states of the Janus In_2_S_2_Se. For *WZ*′ state, the conduction band minimum (CBM) is dominated by the bottom Se layer, the valence band maximum (VBM) is mainly contributed by the top and middle S layers, while under *ZB*′ state, the CBM and VBM are mainly dominated by the top S and bottom Se layers, respectively. Indeed, these distinct charge separation behaviors between the two states are driven by their intrinsic opposite OOP polarization, and further affecting their photovoltaic performances. In Figure 2c,d, the charge density differences between the two states are displayed. Obviously opposite and significantly enhanced charge transfer across the plane can be detected of the *WZ*′ state than *ZB*′, and consistent with the more accurate Bader charge results, where the charge transfer under *WZ*′ and *ZB*′ states are 2.114 and ‐2.107, respectively. So far, the opposite direction and stronger intensity on OOP polarization of the *WZ*′ than *ZB*′ state are confirmed.

**Figure 3:**
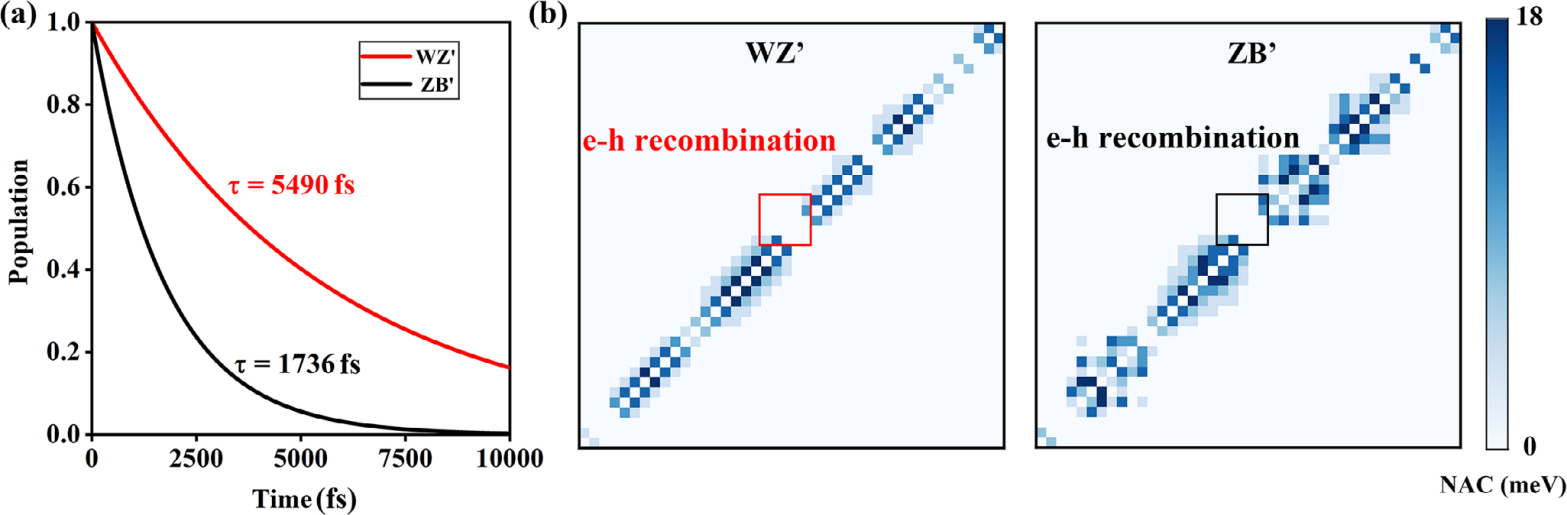
a)The distribution of the carrier population in the *WZ*′ and *ZB*′ states, which is approximately linearly fitted over short‐time linear period by $P\left(t\right)=\exp\;\left(-\frac{t}{\tau }\right)=1-\left(-\frac{t}{\tau }\right)$. b) Distribution of averaged NAC values for the *WZ*′ and *ZB*′ states, with different colors denoting the varying coupling strength.

Building upon the different OOP polarization features, we further explore the distinct photogenerated carrier lifetimes between *WZ*′ and *ZB*′ states. The NAMD were employed to the carrier population evolutions. As shown in **Figure** **3**a, the e‐h recombination times of *WZ*′ phase can be reached 5490 fs, ≈ 3 times larger than that of *ZB*′. Such prolonged lifetimes suggesting more efficient spatial charge separation capability of the *WZ*′ phase. More accurately, the underlying physics between these two evolutions are further captured by the average non‐adiabatic coupling (NAC) matrix elements:

(1) $${\vec{d}}_{ij}=\langle \varphi_{i}|\frac{\partial }{\partial t}\left|\varphi_{j}\right\rangle =\frac{\left\langle \varphi_{i}|\nabla_{R}\hat{H}|\varphi_{j}\right\rangle }{\varepsilon_{j}-\varepsilon_{i}}\dot{R}$$

where $\hat{H}$ denotes the Kohn‐Sham Hamiltonian, φ_*i*/*j*_ and ε_*i*/*j*_ are wave functions and eigenvalues of each electronic states, and $\dot{R}$ indicates the velocity of nuclei. As displayed in Figure 3b, *WZ*′ state exhibits smaller NAC values than *ZB*′ around the bandgap. This is due to the larger vertical dipole, reducing wave‐function overlap and slowing charge transfer, thereby prolonging the e–h recombination for superior photovoltaic performances.

To explore the different carrier mobilities (µ_2*D*_) of monolayer In_2_S_2_Se between *WZ*′ and *ZB*′ states, the µ_2*D*_ of these two states are calculated according to the following expression based on the DP theory, which has been widely used to simulate the µ_2*D*_ of 2D crystals:^[^ ^52^ ^]^

(2) $$\mu_{2D}=\frac{eh^{3}C_{2D}}{k_{B}T\left|m_{e/h}^{*}\right|E_{1}^{2}}$$

where *E*_1_ denotes the variable state, $m_{e/h}^{*}$ is the effective mass of electrons/holes, *T* is the temperature, *k*_*B*_ is the Boltzmann constant, and *C*_2*D*_ indicates elastic modulus for each state crystal. The obtained results are displayed in Table S2 (Supporting Information), where the µ_2*D*_ of Janus In2S2Se are evidently higher than those of monolayer α‐In2Se3,^[^ ^37^ ^]^ indicating its superior photovoltaic performance. More interestingly, since higher µ_2*D*_ of *ZB*′ than *WZ*′ state can be obtained, enhanced photocurrent in it based nano‐device is also predicted. Over all, by expanding monolayer α − *In*_2_ *Se*_3_ to the Janus In_2_S_2_Se, we not only propose a photovoltaic system with superior performances, but can also achieve effective regulation of non‐degenerate sliding ferroelectricity on photovoltaics in a single monolayer system.

### 3.3 Photocurrent Differences between *WZ*′ and *ZB*′ *In*_2_S_2_ *Se* Based Nano‐devices

**Figure 4:**
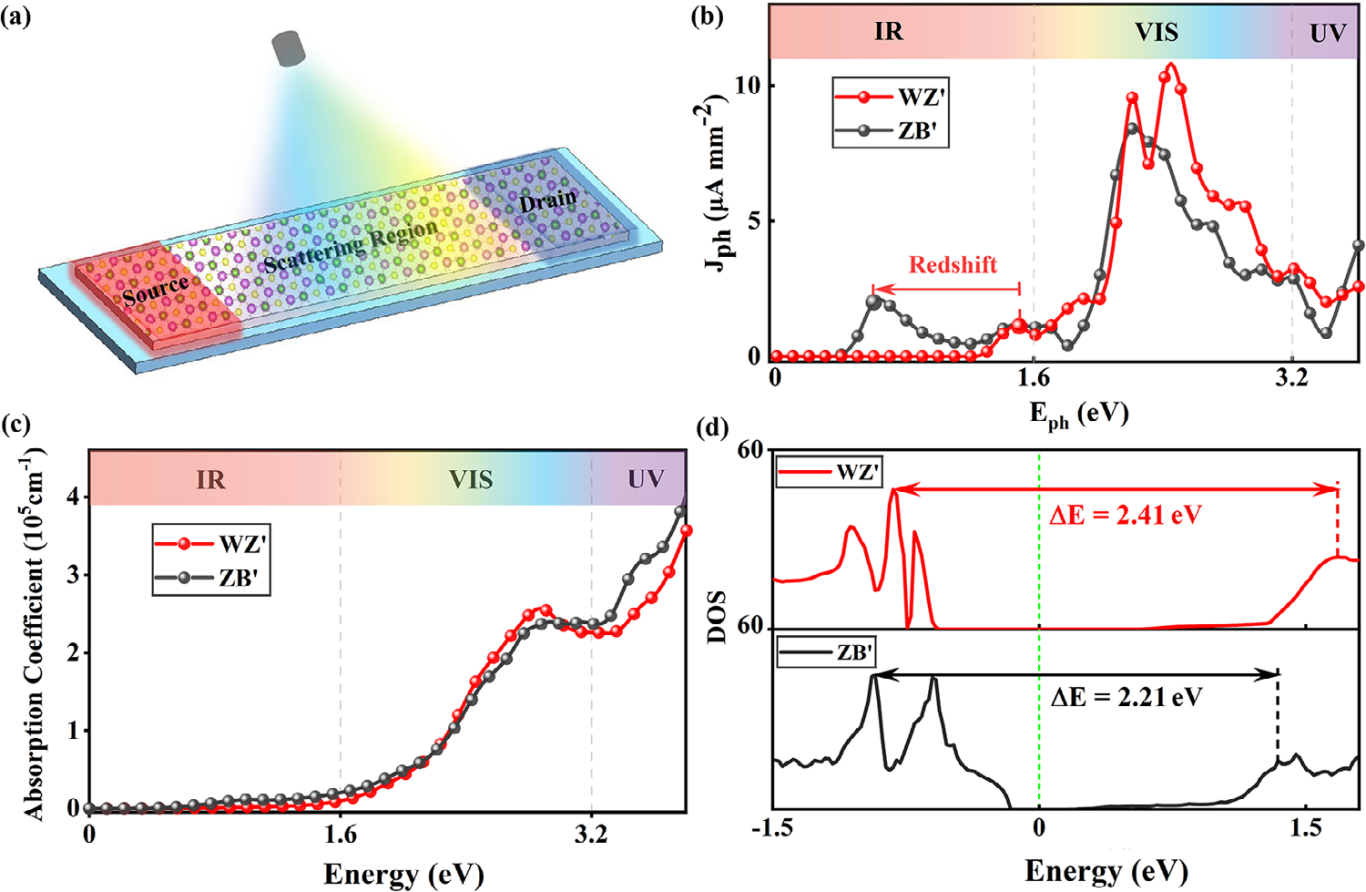
Schematic diagram of the monolayer In2S2Se based two‐probe photoelectric nano‐devices. b) Evolutions of $J_{L}^{ph}$ versus *E*_*ph*_ between the devices under *WZ*′ and *ZB*′ states. c) Evolutions of the optical absorption coefficients *a*(ω) versus Eph between *WZ*′ and *ZB*′ states. In each panel of (b) and (c), the visible light region are distinguished between the two verticEal dashed lines. d) Density of devices states (DOS) Evolutions versus energy between the *WZ*′ and *ZB*′ devices.

Despite excellent photovoltaic characteristics of *WZ*′ and *ZB*′ In_2_S_2_Se have been confirmed, they provide qualitative predictions due to the constraints imposed by periodic boundary conditions. To further explore their actual excellence in practical devices, it is essential to account for quantum scattering under open boundary condition. Therefore, *WZ*′ and *ZB*′ In_2_S_2_Se based nano‐devices are designed according to **Figure** **4**a. For each device, both leads are constructed by periodically extending the scattering region, and such simplified device model has proven to be effective and optimal in a series of previous theoretical and experimental research.^[^ ^53^ ^]^ More accurately, the band structures of the periodic WZ' and ZB' In_2_S_2_Se crystals are also calculated by using Nanodcal package. As displayed in Figure S3 (Supporting Information), almost identical results with VASP results Figure 2a,b can be observed, suggesting their highly consistency and compatibility.

During our calculation, the entire scattering region was illuminated by linearly polarized light perpendicular to the plane. A minute bias voltage of 0.2 eV is applied between source and drain, which is far less than the band gap and only intended to drive the photocurrent. Under the first‐order of Born approximation, the photocurrent ingress into the left probe can be expressed as:^[^ ^54^, ^55^, ^56^ ^]^

(3) $$\begin{array}{c} I_{L}^{ph}=\frac{ie}{h}\int \mathrm{Tr}\left[\Gamma_{L}\left\{G^{<(ph)}+f_{L}\left(E\right)\left(G^{>(ph)}-G^{<(ph)}\right)\right\}\right]dE \end{array}$$

where *f*_*L*_ is the Fermi distribution function of the left lead, *G* ^>/ < (*ph*)^ represents the greater/lesser Green's function, indicating the electron–photon interaction, and Γ_*L*_ is the line‐width function, signifying the coupling between the left lead and the central scattering region. The calculated photocurrent density $J_{L}^{ph}$ = $I_{L}^{ph}/S$ with polarization angle θ = 0° are displayed in Figure 4b. For both states, $J_{L}^{ph}$ start to vibrate when the incident light energy *E*_*ph*_ increases to the value of bandgap, subsequently increasing rapidly. Series of inspiring photovoltaic behaviors can be achieved between these two states. First, significant peak values of $J_{L}^{ph}$ can be obtained under both states, with the magnitudes can be reached 10.79 and 8.42 µ*Amm* ^−2^ for *WZ*′ and *ZB*′ states. Such efficient $J_{L}^{ph}$ peak values are higher than those of monolayer In_2_Se_3_ (3.95 µ*Amm* ^−2^) under the same basis of calculation,^[^ ^57^ ^]^ which is consistent with the above predictions in Figure 2c,d and can be illustrated by the enhanced OOP polarization perfectly. Besides, enlarged $J_{L}^{ph}$ of *WZ*′ than *ZB*′ state can be detected within the visible light region, indicating its higher solar photovoltaic conversion efficiency. To delve into the intrinsic motivations, the light absorption, photogenerated carrier separation and transport characters are compared and analyzed in‐depth. As shown in Figure 4c, the absorption coefficients are approximately consistent throughout the entire light range of E_*ph*_, including the infrared, visible light, and ultraviolet regions, effectively eliminating the influence of light absorption on the difference of $J_{L}^{ph}$ between the two states. In fact, such different solar photovoltaic conversions between *WZ*′ and *ZB*′ states are closely related to their distinct OOP polarization as we have discussed in Figure 1c, where the intensified OOP polarization will drive more efficient separation of photo‐generated carriers, so is the enlarged $J_{L}^{ph}$ in the visible light region. Besides the magnitude of $J_{L}^{ph}$, the E_*ph*_ positions of their peak are also different from Figure 4b, where the peak value of $J_{L}^{ph}$ appears at *E*_*ph*_ = 2.41 eV for *WZ*′ state and 2.21 eV for *ZB*′ state. These different peak positions can be attributed to the distinct density of state between the two devices. As shown in Figure 4d, the *E*_*ph*_ gaps between the two initial peaks near the fermi energy are also 2.41 eV for *WZ*′ state and 2.21 eV for *ZB*′ state. The enhanced density of states can provide favorable guarantees for effective carrier transmission, and ultimately resulting in the peak of $J_{L}^{ph}$. Furthermore, a significantly enlarged and red‐shifted peak value of $J_{L}^{ph}$ is observed in *ZB*′ than *WZ*′ state within the infrared region. The red‐shift phenomenon can be attributed to the reduced band gap in the *ZB*′ state, which facilitates earlier oscillations of $J_{L}^{ph}$. Additionally, the augmented peak value of $J_{L}^{ph}$ is associated with the transition from an indirect to a direct band gap, as well as higher carrier mobilities in the *ZB*′ state relative to the *WZ*′ state.

**Figure 5:**
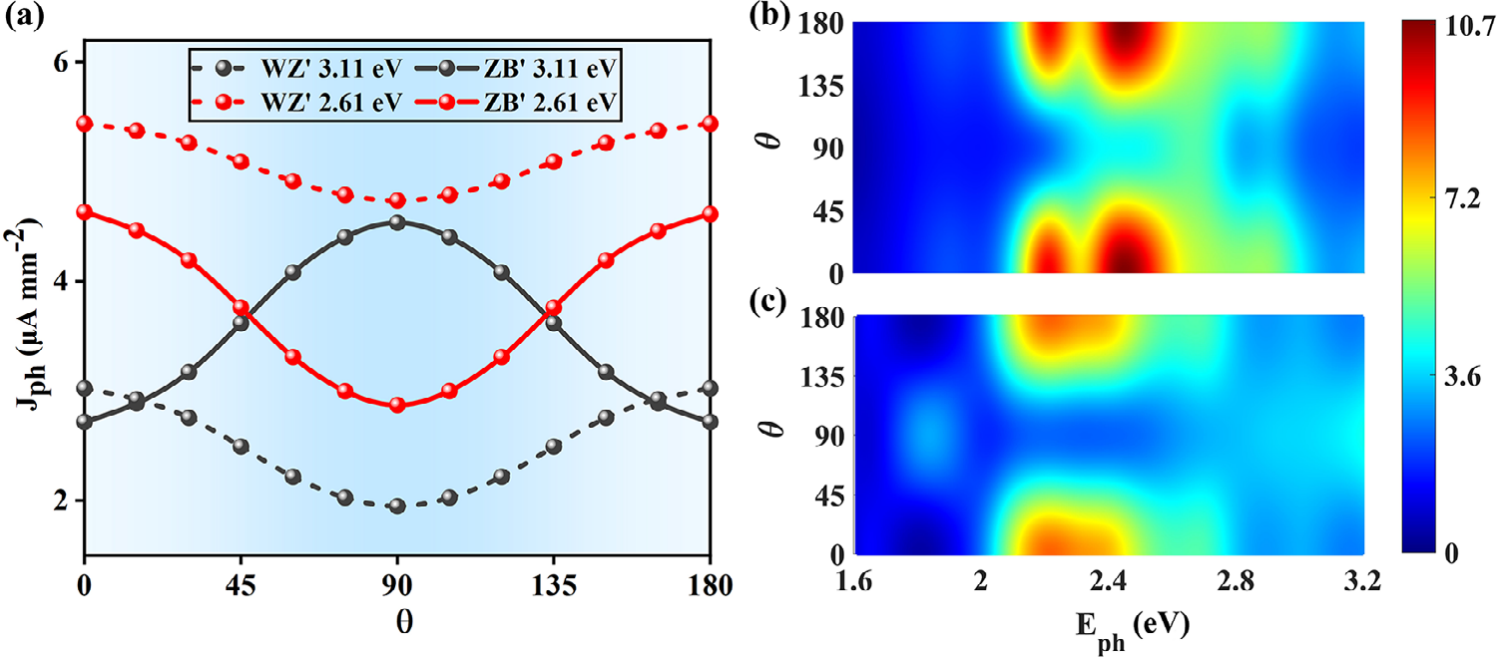
a) The *J*_*ph*_ versus θ at *E*_*ph*_ = 3.11*eV* and *E*_*ph*_ = 2.61*eV* of the *WZ*′ and *ZB*′ state devices. Photocurrent density *J*_*ph*_ versus incident light energy *E*_*ph*_ and polarization angle θ, for *WZ*′ (b) and *ZB*′ (c) state at the PBE level.

Moreover, distinct evolution behaviors of $J_{L}^{ph}$ versus θ are observed for the two phases of Janus In2S2Se, as shown in **Figure** **5**a. When *E*_*ph*_ = 3.11*eV* reversed shapes of $J_{L}^{ph}$ curves can be observed, where $J_{L}^{ph}$ of *ZB*′ state follows the sinusoidal distribution but along a cosine one under the *WZ*′ state; while at *E*_*ph*_ = 2.61*eV*, both curves obey the cosine distributions. These different evolution shapes can be reasonably illustrated from two aspects, one is their distinct structural symmetries according to the different elastic constants in Table S2 (Supporting Information), the other refers to the following reformed expression of $J_{L}^{ph}$ according to Ref.[55]:

(4) $$\begin{array}{cc} J_{L}^{ph}= & \frac{ie}{h}\int \\ & \{cos^{2}\theta \mathrm{Tr}\left[\Gamma_{L}\left\{G_{1}^{<(ph)}+f_{L}\left(E\right)\left(G_{1}^{>(ph)}-G_{1}^{<(ph)}\right)\right\}\right]\\ & +sin^{2}\theta \mathrm{Tr}\left[\Gamma_{L}\left\{G_{2}^{<(ph)}+f_{L}\left(E\right)\left(G_{2}^{>(ph)}-G_{2}^{<(ph)}\right)\right\}\right]\\ & +sin\left(2\theta \right)2\mathrm{Tr}\left[\Gamma_{L}\left\{G_{3}^{<(ph)}+f_{L}\left(E\right)\left(G_{3}^{>(ph)}-G_{3}^{<(ph)}\right)\right\}\right]\\ & \}\times dE, \end{array}$$

where the $J_{L}^{ph}$ is dissected into three components, which are proportional to *sin* ^2^θ, *cos* ^2^θ, and *sin*2θ, respectively. Indeed, the morphological evolution of $J_{L}^{ph}$ bears a profound correlation with the corresponding crystal lattice symmetry, and can be dominated by the coefficients competition between each trigonometric functions. To elucidate further distinctions in solar photovoltaic performance between the *WZ*′ and *ZB*′ phases of In_2_S_2_Se, Figure 5b,c illustrates their θ depended $J_{L}^{ph}$ distributions entire the visible light region. In both states, the maximum peaks of $J_{L}^{ph}$ are located at θ = 0°, accompanied with a marginal red‐shift in peak position occurs in *ZB*′ state. Once again, such distinct peak positions are consisted with the Δ*E* between the two nearest peaks within the device's density of states in Figure 4d. Besides, a notably elevated and broader $J_{L}^{ph}$ peak can be observed in the *WZ*′ state, suggesting its superior efficacy in solar energy conversion compared to the *ZB*′ ones. This enhancement is ascribed to the intensified OOP polarization in the *WZ*′ structure, which finally intensifies the separation of photo‐generated carriers, a pivotal factor in photovoltaic performance. Remarkably, all above distinct excellent characters of $J_{L}^{ph}$ can be regulated via switching the two states of Janus In_2_S_2_Se by sliding ferroelectricity, which is usually easier to be operated than traditional ferroelectrics in experiments.^[^ ^58^, ^59^ ^]^

Finally, based on the distinct photocurrent behaviors between *WZ*′ and *ZB*′ phases, their responsivity *R*_*ph*_ and external quantum efficiency τ_*eqe*_ are further detected for effecting more photovoltaic performances comparison, which can be expressed as:

(5) $$R_{ph}=\frac{J_{ph}}{eF_{ph}}$$

and

(6) $$\tau_{eqe}=\frac{R_{ph}E_{ph}}{e}$$

with *e* denoting the elementary charge, *F*_*ph*_ = *P*_*in*_/(*eE*_*ph*_) indicating the photon flux, and the incident power density being set to *P*_*in*_ = 10^3^ µ*Wmm* ^−2^ to match the typical solar irradiation. The obtained results of *WZ*′ and *ZB*′ phases can be reached 0.025 and 0.020 A W^−1^, higher than those of monolayer *MoS*_2_ (0.016 A W^−1^)^[^ ^60^ ^]^, monolayer graphene (5 × 10^−4^ A W^−1^)^[^ ^61^ ^]^, and *MoS*_2_/*MoS*_2_ heterostructure (0.010 A W^−1^)^[^ ^62^ ^]^ devices. Once again, these ideal performances suggest powerful potential applications of the Janus In_2_S_2_Se monolayer in optoelectronics and photovoltaics, the higher *R*_*ph*_ and τ_*eqe*_, together with the enhanced *J*_*ph*_ in the visible light region of *WZ*′ phase also indicating superior photovoltaic characters than that of the *ZB*′ phase.

## 4 Conclusion

In summary, to address the limitations of recent 2D sliding ferroelectrics in photovoltaic enhancements, we have proposed Janus *In*_2_ *S*_2_ *Se* as a robust platform for establishing effective modulations of photovoltaics via non‐degenerate sliding ferroelectricity. Due to the increased asymmetry of surface atoms, two distinct states and stronger OOP dipoles can be realized in this Janus *In*_2_ *S*_2_ *Se* than monolayer α − *In*_2_ *Se*_3_, thereby behaving superior and controllable photovoltaic characteristics. We show that both states of this Janus *In*_2_ *S*_2_ *Se* are experimentally feasible, and can be switchable via low–barrier (≈ 60*meV*) interlayer sliding. When compared to the conventional α − *In*_2_ *Se*_3_ monolayer, stronger OOP polarization, higher carrier mobility, more efficient light absorption and lower exciton binding energy can be detected in Janus *In*_2_ *S*_2_ *Se*, hence enhanced $J_{L}^{ph}$ can be obtained in their based nano‐devices. Besides, we have also demonstrated the state–dominant modulation of photovoltaic properties in this new predicted Janus *In*_2_ *S*_2_ *Se*. The *WZ*′ to *ZB*′ transition increases carrier mobilities, reduces the photogenerated carrier lifetimes and shifts the bandgap from indirect toward direct character with more moderate size, producing a pronounced red‐shift and enhanced $J_{L}^{ph}$ in the infrared spectrum. Conversely, due to the enhanced OOP polarization in *WZ*′ state, superior $J_{L}^{ph}$ in the visible light region is delivered during the *ZB*′ to *WZ*′ transition, indicating its more efficient solar photovoltaic conversions. Overall, by leveraging state–engineered sliding ferroelectricity in Janus *In*_2_ *S*_2_ *Se*, we introduce feasible physical correlations between sliding ferroelectricity and photovoltaics, and the insight mechanisms may shed new light on the conception and modulation of next‐generation ultrathin and switchable photovoltaic devices.

## Conflict of Interest

The authors declare no conflict of interest.

## Supporting information

Supporting Information

## Data Availability

The data that support the findings of this study are available in the supplementary material of this article.
