# Supplementary material for: Phase‐Engineered Non‐Degenerate Sliding Ferroelectricity Enables Tunable Photovoltaics in Monolayer Janus In2S2Se
Source: Adv Sci (Weinh). 2025 Dec 14;13(11):e20732. doi: 10.1002/advs.202520732 (PMC12931245; doi:10.1002/advs.202520732)
Supplement: Supplementary file 1 — Supporting Information [file ADVS-13-e20732-s001.pdf]

Supporting Information for:

Phase-Engineered Non-degenerate Sliding Ferroelectricity Enables Tunable Photovoltaics in Monolayer Janus In_2S_2Se

Yixuan Li[a] , Qiang Wang[a*], Keying Han[a] , Yitong Liang[a] , Kai Kong[a] , Yan Liang[b] , Thomas Frauenheim[c] , Xingshuai Lv[b] , Defeng Guo[a], Bin Wang[e*]

[a] *State Key Laboratory of Metastable Materials Science and Technology and Key Laboratory for Microstructural Material Physics of Hebei Province, School of Science, Yanshan University,Qinhuangdao, 066000, People's Republic of China.E-mail: qiangwang@ysu.edu.cn*

[b] *College of Physics and Optoelectronic Engineering, Faculty of Information Science and Engineering, Ocean University of Chinay, Qingdao, 266100, People's Republic of China.*

[c] *School of Science, Computational Science and Applied Research Institute (CSAR), Beijing Computational Science Research Center (CSRC) , Constructor University, Bremen, Shenzhen, Beijing, 28759, 518110, 100193, Germany, P. R. China, P. R. China.*

[d] *College of Chemistry and Chemical Engineering, Ocean University of China, Qingdao 266100, P. R. China.*

[e] *Shenzhen Key Laboratory of Advanced Thin Films and Applications, College of Physics and Optoelectronic Engineering, Shenzhen University, Shenzhen, 518000, People's Republic of China. E-mail: binwang@szu.edu.cn*

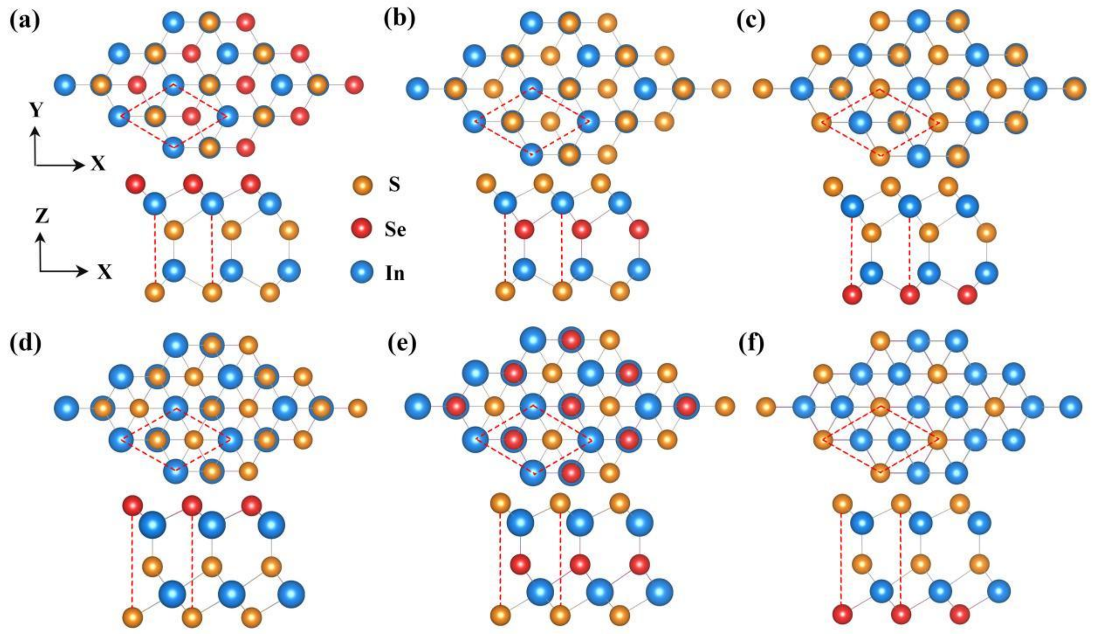

Fig. S1. Top and side views of schematic structures for the distinct substitutions of Janus In_2S_2Se monolayer under WZ' (a-c) and the ZB' (d-f) phases. In each phase, the configurations include t-In_2S_2Se, m-In_2S_2Se, and b-In_2S_2Se, with Se atoms substituting the top, middle, and bottom S layers, respectively.

Table S1. The binding energies (E_b) of the Janus In_2S_2Se and In_2Se_3 are calculated for each structural configuration in both the WZ′ and ZB′ phases.

E_b ($J\cdot m^2$)	t-In_2S_2Se	m-In_2S_2Se	b-In_2S_2Se	In_2Se_3
WZ'	-1.1704	-1.1672	-1.1723	-0.9799
ZB'	-1.1194	-1.1138	-1.1207	-0.9787

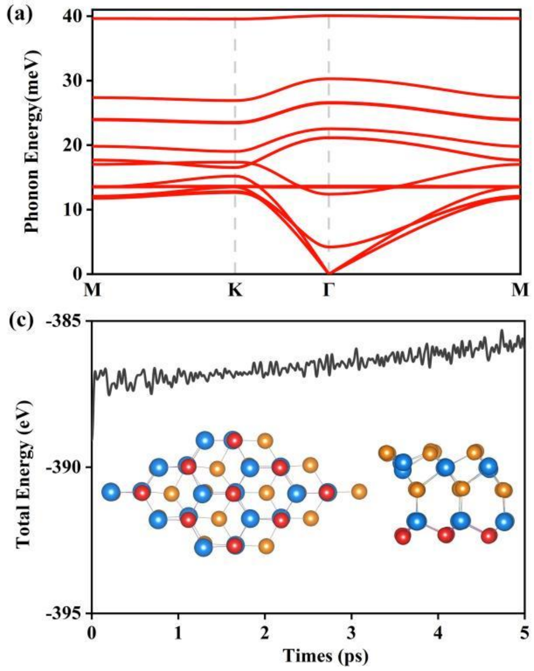

Fig. S2. (a-b) Phonon dispersion curves of the b-In_2S_2Se monolayer under the WZ’ (a) and ZB’ (b) phases. (c-d) Time evolution of the total energy for a 3×3 supercell of the b-In_2S_2Se under the WZ’ (c) and ZB’ (d) phases at 300 K. Insets: Top and side views of representative atomic configurations after 5 ps of molecular dynamics simulation.

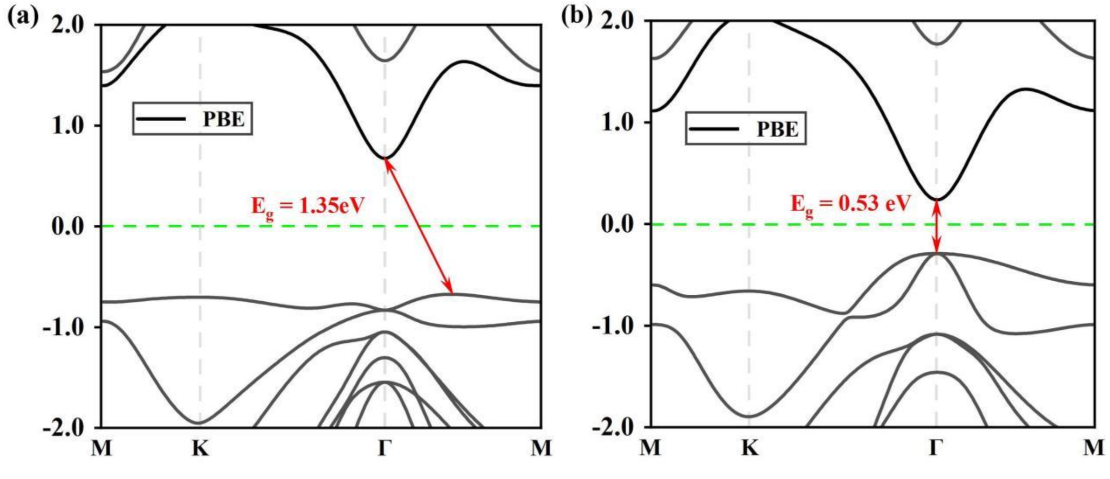

Fig. S3. (a-b) The energy band structures of the WZ′ and ZB′ phases of In_2S_2Se calculated by NanoDcal.

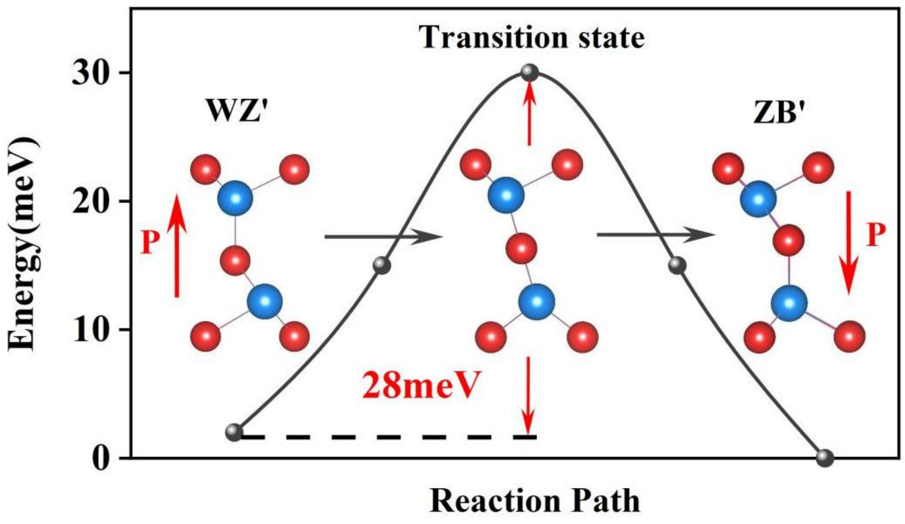

Fig. S4. Energy variation of In_2Se_3 along the out-of-plane ferroelectric switching pathway under NEB simulation. The three inserts depict configurations at the initial, middle, and final states, with P indicating the corresponding OOP polarization strength.

Table S2. Effective mass (m*), elastic modulus (C), deformation potential (E_d), and carrier mobilities (μ_{2D}) between In_2S_2Se and In_2Se_3 monolayers.

Structure		m^* (m_0)		C (Nm^{-1})	E_d (eV)		μ_{2D} ($cm^2V^{-1}s^{-1}$)	
		e	h	e=h	e	h	e	h
In_2S_2Se(WZ')	x	0.36	4.17	92.14	1.84	1.47	2995.07	34.76
	y	0.58	3.51	105.96	3.54	0.46	350.78	586.40
In_2S_2Se (ZB')	x	0.37	3.47	101.77	0.76	1.71	5045.99	40.98
	y	0.37	3.47	110.57	2.99	1.99	317.88	107.19
In_2Se_3 (WZ')	x	0.30	2.51	105.93	2.40	3.07	2958.69	25.29
	y	0.54	2.39	122.33	2.95	0.71	657.34	596.09
In_2Se_3 (ZB')	x	0.29	2.71	103.42	2.02	3.08	4244.27	20.98
	y	0.59	1.92	103.42	3.18	0.95	164.25	219.93
